# Whole-Body Dynamic Positron Emission and Computed Tomography (WBD-PET/CT): Latest Developments, Challenges and Opportunities

**DOI:** 10.3390/diagnostics16121866

**Published:** 2026-06-16

**Authors:** Anastasios Vatalis, Dimitra Tsivaka, Varvara Valotassiou, Emmanouil Panagiotidis, Panagiotis Georgoulias, Nicolas A. Karakatsanis, Ioannis Tsougos

**Affiliations:** 1 Medical Physics Department, Faculty of Medicine, School of Health Sciences, University of Thessaly, 41500 Larissa, Greece; avatalis@uth.gr (A.V.); dtsivaka@uth.gr (D.T.); 2 Department of Nuclear Medicine, Faculty of Medicine, School of Health Sciences, University of Thessaly, 41500 Larissa, Greece; valotasiou@uth.gr (V.V.); manospanagiotidis@uth.gr (E.P.); pgeorgoul@uth.gr (P.G.); 3 Department of Radiology, Weill Cornell Medical College, New York, NY 10021, USA

**Keywords:** dynamic PET/CT, digital PET/CT, radio-pharmacokinetics, theranostics

## Abstract

Whole-body dynamic positron emission tomography/computed tomography (WBD-PET/CT) has transformed medical imaging, enabling the fusion between (i) detailed anatomical maps of the human body and (ii) quantitative multi-parametric functional maps of specific biochemical and physiological processes across the human body beyond the semi-quantitative limitations of static PET/CT imaging. Latest developments in systems hardware, particularly with the introduction of long-axial-field-of-view (LAFOV) and Time-of-Flight (TOF) PET scanners and low-dose CT scanners, and in data analysis, primarily with direct parametric PET image reconstruction and Artificial Intelligence, offer unprecedented opportunities towards the wide clinical adoption of the superior quantitative accuracy and precision of WBD-PET/CT imaging overcoming current challenges, such as data acquisition complexity and long scan durations. This review aims to summarize the latest developments, current challenges, and emerging opportunities in WBD-PET/CT, emphasizing its potential to broaden the diagnostic and theranostic role of PET/CT in clinical practice.

## 1. Introduction

Positron Emission Tomography (PET) and Computed Tomography (CT) are two advanced medical imaging techniques whose combination has revolutionized medical diagnostics and more recently personalized radionuclide treatments. PET imaging provides detailed and unique functional information by detecting gamma rays emitted from a radiotracer injected into the bloodstream and specifically interacting with tissues according to its molecular profile offering valuable insights into the molecular functionality of healthy and diseased tissues for either diagnosis or personalized therapy [1]. CT, on the other hand, generates detailed anatomical images of the tissues’ electron density properties by measuring the attenuation properties of X-rays passing through the human body at multiple angles. When combined, hybrid PET/CT imaging merges the functional maps from PET with the structural maps of CT, enabling clinicians to obtain a more comprehensive anatomically referenced map of specific molecular processes throughout the human body [2].

The development of hybrid PET/CT imaging began in the early 2000s, marking a significant milestone in medical imaging. Previously, PET and CT were performed separately, often requiring patients to undergo multiple scans, resulting in longer exam times and a higher risk of misalignment between functional (PET) and anatomical (CT) images [3]. The integration of PET and CT into a single machine has addressed these issues, improving the accuracy and efficiency of diagnosis, particularly in oncology, cardiology, and neurology. By providing simultaneous visualization of both the metabolic activity and anatomical structure of tissues, PET/CT has become a cornerstone in the staging, treatment planning, and monitoring of various diseases, especially cancer [4].

Advancements in PET/CT technology have been driven by improvements in scanner sensitivity, spatial resolution, advanced quantitative imaging data analysis and the development of novel radiotracers efficiently targeting specific molecular processes associated with important physiologic and disease-related mechanisms, including metabolism, cell proliferation, hypoxia, inflammation, perfusion, neuronal activity, etc. Innovations in CT technology, such as multi-detector arrays and lower radiation dose protocols, have also enhanced the quality of PET/CT imaging [5]. Additionally, the growing use of dynamic PET/CT, which tracks changes in metabolic activity over time, has expanded its applications in lesion detectability and uptake quantification, treatment response monitoring, disease progression evaluation and safe and efficacious personalized radionuclide treatment planning. Beyond oncology, cardiology, and neurology, PET/CT is now being used in fields like immunology and infectious diseases. Moreover, recent advances in simultaneous PET/MRI, synthesis of tracers targeting immune checkpoint pathways and development of theranostic imaging agents labelled with alpha, beta or gamma radioisotopes for personalized image-guided radionuclide treatments are revolutionizing the diagnostic and therapeutic horizons [6].

### Methodological Approach

For this narrative review, literature was primarily retrieved from PubMed and Google Scholar databases covering the period from 2010 to 2025, with emphasis on recent developments in dynamic whole-body PET/CT, long-axial-field-of-view (LAFOV) PET systems, kinetic modeling, parametric imaging, and AI-assisted image reconstruction. Search terms included combinations of ‘dynamic PET/CT’, ‘whole-body dynamic PET’, ‘LAFOV PET’, ‘parametric imaging’, ‘kinetic modeling’, ‘Patlak analysis’, ‘Logan analysis’, and ‘theranostics’. Priority was given to peer-reviewed original articles, major review papers, and clinically validated technical studies directly relevant to the topic. Studies were selected based on methodological relevance, clinical applicability, and contribution to the understanding of current challenges and future opportunities in WBD-PET/CT.

## 2. Advantages of Digital and LAFOV PET/CT

Digital PET/CT systems are a groundbreaking advancement in medical imaging, offering enhanced capabilities through the integration of fast silicon photomultipliers (SiPMs), advanced time-of-flight (TOF) detector technology and faster electronic readout circuits. Unlike analog systems, which rely on photomultiplier tubes (PMTs), digital systems with fast SiPMs and readout, not only enhance spatial resolution but also enable higher TOF measurement precision to enhance effective sensitivity translating to higher signal-to-noise ratio (SNR) [7]. These features enable better visualization of small lesions, enhanced contrast in images, and more precise quantification of tracer uptake, making digital PET/CT particularly valuable in oncology, neurology, and cardiology [8].

Furthermore, they can drastically improve lesion detectability, particularly in cases involving large patients or low-dose imaging scenarios, thereby broadening their clinical and research applications [9]. In addition, digital PET/CT’s high SNR enables capturing faster temporal changes in tracer kinetics in blood and tissues, providing valuable quantitative tracer kinetic parameters such as blood flow, tissue binding or absorption and washout rates for more accurate differential and multi-parametric diagnostic and therapy response assessments. These attributes enhance the ability to differentiate malignant from benign or physiological non-specific signal, improve treatment planning, evaluate systemic diseases, and conduct advanced kinetic modeling, which are increasingly important in precision medicine [10].

The long axial field-of-view (LAFOV) capabilities of modern PET/CT systems allow for whole-body imaging in a single or much fewer bed positions, compared to short axial field-of-view (SAFOV) scanners, thus permitting truly simultaneous data acquisition protocols and the inevitable temporal gaps in acquisition for both static and dynamic scans [11]. Furthermore, multi-organ axis studies may now be possible between distant and seemingly unrelated organs, such as the real-time molecular associations between brain and heart or gut microbiome functions after a triggering event [12].

Moreover, the higher solid-angle coverage of LAFOV allows for multi-fold higher scan sensitivity translating to significantly reduced static acquisition times and improved static patient exams throughput for the same amount of injected dose relative to SAFOV systems [13]. In the case of dynamic scans, the higher sensitivity can benefit temporal resolution, similarly to and in addition to TOF’s enhanced effective sensitivity, for tracking also the more rapid components of certain tracer kinetics. Equivalently, LAFOV PET/CT scanners can tolerate significantly lower injected doses without compromising diagnostic quality for the same acquisition time as SAFOV systems, therefore considered particularly advantageous in paediatric imaging, screening studies and high-risk populations [14].

**Figure 1:**
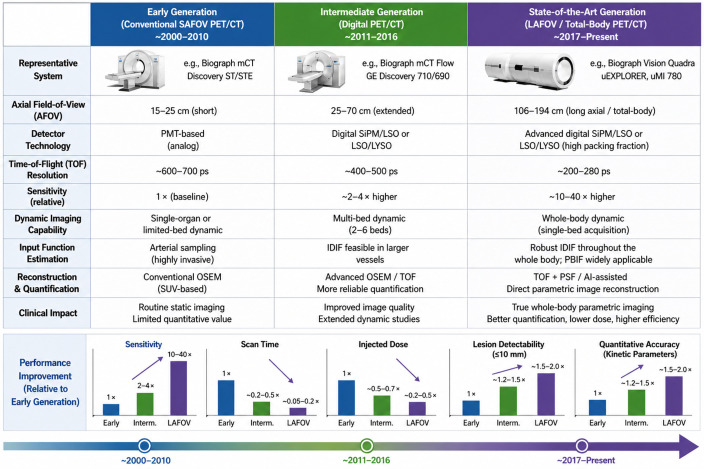
Evolution of Whole-Body Dynamic PET/CT technology from early conventional short axial field-of-view (SAFOV) systems to state-of-the-art long axial field-of-view (LAFOV) and total-body PET/CT platforms. The figure illustrates the progressive improvements in hardware performance, including axial field-of-view, detector technology, time-of-flight (TOF) resolution, sensitivity, and dynamic imaging capability, as well as advances in input function estimation, reconstruction methods, and clinical applicability. Compared with early-generation systems, modern LAFOV PET/CT enables true whole-body single-bed dynamic acquisition, significantly reduced scan duration and injected dose, improved lesion detectability, and enhanced quantitative accuracy through direct parametric imaging and more robust kinetic modeling, supporting the transition from static semi-quantitative imaging toward fully quantitative multi-parametric whole-body PET imaging.

The evolution of WBD-PET/CT can be broadly divided into three developmental stages, as shown in Figure 1. Early-generation systems were constrained by limited axial field-of-view, lower sensitivity, and analog detector technology, restricting dynamic imaging mainly to single-organ studies [15,16]. Intermediate systems introduced digital detector technology, improved TOF resolution, and more advanced reconstruction algorithms, enabling multi-bed dynamic acquisitions and improved lesion detectability. Contemporary LAFOV PET/CT systems now allow true whole-body single-bed dynamic imaging with significantly enhanced sensitivity, more reliable image-derived input functions, and direct parametric image reconstruction, substantially improving quantitative accuracy, scan efficiency, and theranostic potential [17]. These advances collectively represent a major shift from semi-quantitative static imaging toward fully quantitative multi-parametric whole-body imaging [18].

Recent studies have shown that LAFOV PET/CT systems can reduce acquisition times by approximately 5–10-fold compared to conventional short axial field-of-view (SAFOV) scanners while preserving comparable diagnostic image quality, depending on the clinical indication and protocol design [19]. Similarly, administered radiotracer activity reductions of 50–80% have been reported, particularly in pediatric imaging, screening studies, and serial follow-up examinations, where minimizing radiation exposure is particularly important. Ultra-low-dose total-body dynamic PET studies have demonstrated kinetic parameter estimation and lesion detectability comparable to standard full-dose protocols [2].

In digital PET/CT, improved TOF resolution and higher sensitivity have been associated with enhanced lesion detectability, particularly for sub-centimeter lesions and in obese patients [8]. Several studies report improvements in contrast recovery and lesion detection rates ranging from 15% to 30%, depending on lesion size, tracer kinetics, and acquisition parameters [20]. These improvements are particularly relevant in oncologic imaging, where small lesion detection directly affects staging accuracy and treatment planning.

## 3. Key Contributions of Dynamic PET/CT

Dynamic and static PET/CT imaging differ fundamentally in their approach and the type of information they provide. Static PET/CT involves capturing a single snapshot of radiotracer distribution at a specific time point after injection. This method is simpler, faster, and widely used clinically for tasks such as tumor detection, staging, and monitoring response to therapy, relying heavily on semi-quantitative metrics like the standardized uptake value (SUV) [21]. Dynamic PET/CT, on the other hand, involves time-segmented imaging after the tracer injection and over the course of its circulation in blood, uptake in tissues and clearance, providing detailed kinetic data. This allows tracking of the tracer absolute activity concentration in both the blood and tissues over post-injection time to enable the calculation of highly quantitative specific physiological parameters related to the tracer kinetics [22] accounting for the tracer availability in blood and its kinetics, unlike the semi-quantitative SUV metric in static imaging. Depending on the assumed kinetic model and the uptake mechanism for a given tracer and tissue combination, the tracer kinetic parameter definitions can vary, such as blood flow, perfusion, metabolic rates, receptor binding potential etc. [23].

**Figure 2:**
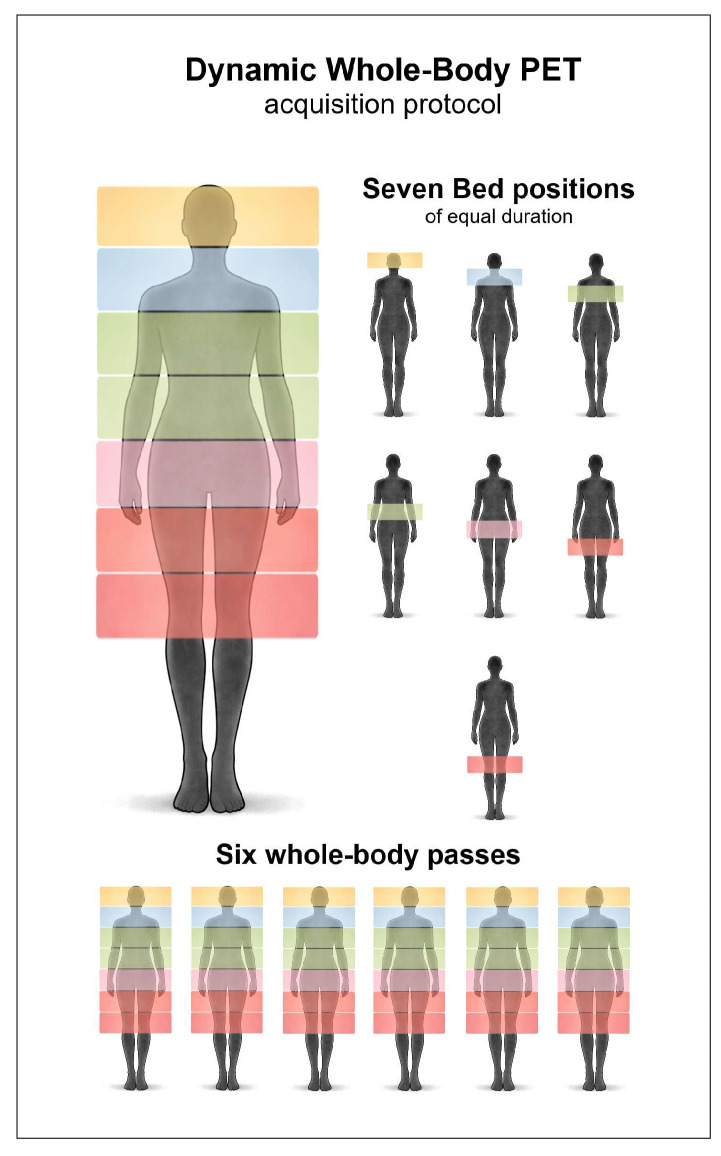
Schematic representation of the acquisition sequence during the second phase of a step-and-shoot dynamic whole-body PET/CT protocol. Six consecutive cranio-caudal whole-body passes are performed, each consisting of seven bed positions of equal duration. The colored horizontal bands are used only to visually distinguish the successive axial bed positions along the patient’s body and do not represent different acquisition parameters, tracer uptake levels, or reconstruction settings.

A schematic representation is shown in Figure 2, which depicts the full sequence of dynamic bed positions obtained using the step-and-shoot mode during the second phase of the DWB acquisition. Six unidirectional whole-body (cranio-caudal) passes are performed, each consisting of seven bed positions with identical acquisition times [24].

Dynamic imaging offers richer quantitative insights relative to static imaging but requires more complex acquisition protocols, longer scan times to sufficiently capture all different tracer kinetic components, high temporal resolution to ensure enough count statistics per time frame and advanced computational tools for kinetic data analysis [15]. While static imaging is adequate for many diagnostic purposes, dynamic imaging is especially valuable in research and precision medicine, enabling the differentiation of pathophysiological processes, such as distinguishing aggressive from indolent tumors or more accurately assessing treatment response at a metabolic level [25] by eliminating potential confounding effects such as variable scan time windows between exams, tracer metabolites in blood plasma and sub-optimal tracer injections causing dose leakage outside the veins at injection site and thus less administered dose amounts than expected. These limitations highlight the important role of dynamic and multi-parametric PET imaging as a complement to static SUV PET imaging, tailored for cases where deeper physiological insights are necessary [23].

Dynamic PET/CT imaging is a critical area of research in nuclear medicine, offering enhanced diagnostic capabilities and kinetic data that can improve disease characterization, therapy planning, and treatment monitoring. In the following sections we present selected fields where dynamic PET has already demonstrated significant contributions or potential for the near future:

1. Enhanced Diagnostic Power

Whole-body dynamic PET/CT represents a significant leap in medical imaging, providing comprehensive insights into various aspects of multi-organ molecular interactions across the human body through tracer-specific kinetic modeling. By capturing time-dependent changes in tracer’s body distribution post-injection, this technology allows for a more in-depth and quantitative characterization of both normal physiological as well as abnormal pathological processes for improved diagnostic assessments at an earlier stage [18].

In oncology, this innovation may enable clinicians to distinguish aggressive tumors from indolent ones by quantifying metabolic rates or receptor-binding rates extracted from dynamic scans to complement the semi-quantitative time-averaged measurements of the metabolized or bound tracer amount in tissue extracted from static scans. For instance, tumors with high metabolic activity typically demonstrate throughout the post-injection period a constantly elevated uptake rate of FDG (fluorodeoxyglucose), a glucose analog [10]. On the other hand, static PET scans can only assess a time snapshot of the tracer activity concentration at a specific time window which may considerably vary as a result of the dynamic kinetics of the tracer in the body. Beyond simply identifying lesions via their uptake contrast relative to their surrounding background at a specific time window post-injection, whole-body dynamic PET/CT provides more consistent quantification of the actual rate of the tracer uptake over a larger post-injection time window, thereby enhancing lesion detectability compared to static PET/CT imaging. This improved resolution and precision contributes to more accurate staging and treatment planning [26].

Moreover, the ability to assess the tracer uptake dynamics, including its uptake and wash-out rates, simultaneously at different organs across the human body before or after a trigger could provide valuable insights into the functional associations across different organs enabling the more in-depth study of local and systemic diseases [27]. For example, it can elucidate the relationship between cardiovascular and neurologic inflammation, gut microbiota characteristics and brain health, cardiac function and renal perfusion or any correlations between systemic inflammation and the spread and metabolic activity of cancer metastases throughout the body [12].

2. Total-Body PET/CT Applications

Long-axial-field-of-view (LAFOV) PET/CT scanners are at the forefront of imaging innovation, offering unparalleled sensitivity and speed. These scanners can cover the entire human body (with 2 m AFOV) or torso (with 1 m AFOV) in a single acquisition at a larger solid-angle coverage to achieve multi-fold enhanced sensitivity, therefore dramatically increasing the rate of counts acquired at a given time and thus enabling the dramatic reduction of scan times or administered doses [28]. The scan time reduction for a certain administered dose can be directly translated to minimizing patient discomfort, further limit radiation exposure for patients and staff and increase daily exams throughput while enabling scans entirely in breath-hold mode to mitigate motion effects or fast pediatric exams without the need for anesthesia. On the other hand, reducing administered dose for a certain scan duration would allow scanning more sensitive or vulnerable populations if needed, including pediatric and geriatric patients, reduce exposure of patients in longitudinal studies where multiple exams are involved, or conducting PET imaging screening studies in larger populations to enable detection of diseases at earlier stages or better characterize healthy metabolic function [8,29,30].

In cardiology, dynamic PET imaging can enable detailed analyses of myocardial perfusion and ejection fraction, critical for diagnosing ischemic heart disease [31], for qantitative assessment of cardiac sarcoidosis [32,33] or assist with PET attenuation correction in absence of CT [34]. While attenuation correction is typically performed using CT-derived attenuation maps, dynamic PET data may indirectly support alternative or complementary attenuation correction strategies, particularly in cases where CT data are unavailable, limited, or affected by motion. For example, dynamic acquisitions can facilitate data-driven approaches to segment the bone tissue and include them as an additional tissue class in PET attenuation maps [34] or to assist motion tracking and correction techniques that improve the spatial alignment and quantitative accuracy of PET measurements [35].

Furthermore, LAFOV PET/CT systems offer the ability to obtain more reliable image-derived input functions from larger blood pools included in their axial FOV (e.g., left heart ventricle and aorta), compared to the smaller carotid arteries included in the limited axial FOVs centered at the brain, to enable more accurate kinetic parameter estimates in brain regions [36,37]. Similarly, in systemic diseases such as inflammatory or autoimmune disorders, total-body dynamic PET/CT can provide valuable insights into the extent and the specificity of inflammation with respect to normal and abnormal body functions by tracking first-order kinetics of inflammation tracers across multiple organs simultaneously [38].

3. Advances in Kinetic Modeling and Parametric Imaging

Dynamic imaging has catalyzed the development of advanced kinetic modeling and parametric imaging techniques utilizing the extra dimension of time in the measurements to extract additional information about the tracer properties that have clinical value. Traditional PET imaging often relies only on static 3D measurements of the semi-quantitative metric of the Standardized Uptake Value (SUV) which is defined as the tracer activity concentration at a certain time post-injection normalized to the ratio of the administered dose over the body weight. SUV is considered a semi-quantitative metric as it only provides a snapshot of the tracer uptake at a single time point relative to injection assuming all administered dose entered the arterial blood circulation and is available for uptake by the tissues [39,40].

**Figure 3:**
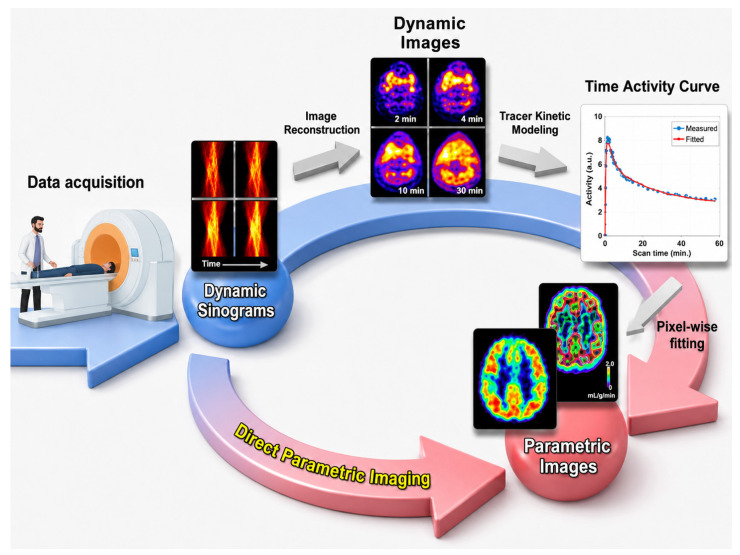
Parametric imaging for tracer kinetics is typically illustrated by two approaches: an indirect method based on kinetic modeling of reconstructed dynamic images, and a direct method that derives parametric images directly from raw projection data.

Time-activity curve (TAC) analysis is central to these research efforts, providing a quantitative framework for evaluating tissue response and absorbed dose over time [41]. In Figure 3, a visual representation of parametric imaging for tracer kinetics typically highlights two approaches. The indirect method involves reconstructing dynamic images first, followed by applying tracer kinetic modeling to these images [42]. In contrast, the direct method derives parametric images directly from the raw projection data without intermediate reconstruction steps. For instance, TACs can reveal differences in radiotracer uptake patterns between normal and diseased tissues, contributing to the early detection of pathologies or the identification of novel therapeutic targets while they can also be utilized to more accurately assess cumulative absorbed dose at the voxel level [43].

However, these assumptions may not be always very accurate while the static measurements largely depend on the post-injection time window. Therefore, any real effects on static SUV measurements may be confounded by one of the above factors diminishing the full quantitative value of PET imaging [8]. On the contrary, dynamic PET imaging enables the measurement of the activity concentration in tissue and blood as a function of post-injection time to produce tissue time-activity curves (TACs) and arterial blood input functions, which can then be fitted to robust graphical analysis models or high-order compartmental kinetic models to offer more quantitative metrics of the tracer uptake features accounting for the tracer availability in blood and its absorption and wash-out rate from tissue and ultimately allowing for more reliable tumor staging, differentiation from normal physiological uptake and more accurate assessment of actual treatment response free of the SUV confounding factors [44].

Graphical analysis methods, such as Patlak and Logan analysis [24,26,45,46], uses linearized equations to describe the macro-kinetic effects of the tracer activity concentration in tissues and they are known for their robustness and simplicity. Furthermore, tracer kinetic modeling uses higher order tissue compartmental models to describe the micro-kinetic behavior of tracers at a higher detail. For instance, in the case of FDG a two-tissue compartmental model is typically employed to describe the kinetics of FDG irreversible metabolism in the cell. Once inside most types of healthy tissue or abnormal cells, FDG is irreversibly phosphorylated to form 18F-FDG-6-phosphate, effectively trapping the radiotracer and providing a marker for glucose metabolism [47]. This model accounts for rate constants such as K1 (tracer transport into the cell), k2 (tracer transport out of the cell), and k3 (phosphorylation rate). Although the dephosphorylation rate constant, k4, is typically negligible rendering the metabolic uptake process effectively irreversible and simplifying the complexity of the differential solving equations [48], several studies have suggested [49,50] that certain organs, such as the liver, and tumor types, such as the hepatocellular carcinoma (HCC) exhibit non-negligible k4 rates, therefore requiring more complex graphical analysis or tissue compartmental models to attain higher accuracy by accounting for these effects [51,52].

**Figure 4:**
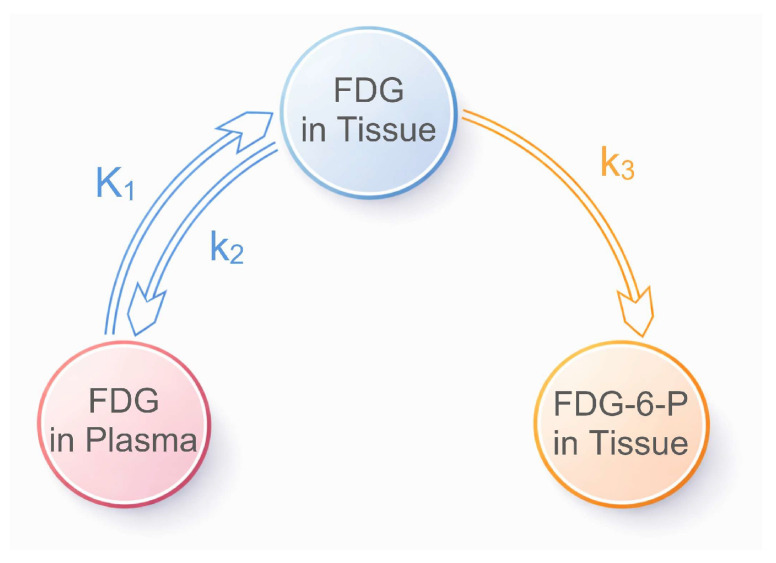
The commonly used two-tissue compartmental model for FDG assumes cellular uptake and phosphorylation that effectively trap the tracer, with the reverse rate (k4) usually negligible—except in normal liver and some tumors—making the process largely irreversible and applicable to similar radiotracers.

Parametric imaging relies on kinetic modeling to quantify the various tracer kinetic parameters at the voxel level, eventually generating parametric maps of specific and highly quantitative physiological parameters, such as blood flow and perfusion, metabolic rate, wash-out rate, or receptor binding potential [53]. These maps offer a richer dataset for more precise clinical interpretations, potentially allowing for the more distinct differentiation of tumor malignancy vs. benignancy, tumor grading or the assessment of treatment efficacy [28]. As shown in Figure 4, a commonly used two-tissue compartmental model for FDG (fluorodeoxyglucose) assumes that FDG, a glucose analog, is absorbed by cells with high glucose metabolism [28]. Inside the body, FDG undergoes phosphorylation to form 18F-FDG-6-phosphate, effectively trapping the radiotracer within the cell. The reverse process, represented by k4 (the backward rate constant opposing k3), is generally considered negligible for most tissues in the case of FDG, making the last step effectively irreversible and simplifying solution complexity. This model is also applicable to other radiotracers that exhibit similar biological behavior. Note that k4 should not be omitted in case of normal liver and certain tumor FDG uptake kinetics as its neglect may lead to bias in the parameter estimation [28].

Patlak graphical analysis is particularly suitable for irreversible tracers such as 18F-FDG, where intracellular trapping enables robust estimation of the net influx constant (Ki) using shortened dynamic acquisition protocols and simplified input function strategies [54]. Logan analysis is more appropriate for reversible tracers, especially in neuroreceptor imaging, where reversible ligand binding dominates tracer kinetics. Full compartmental modeling remains the preferred method when detailed micro-kinetic characterization is required, although it requires longer acquisition times, higher temporal resolution, and more complex parameter estimation [18].

For clinical implementation, image-derived input functions (IDIFs) and population-based input functions (PBIFs) are increasingly used to reduce the need for invasive arterial sampling and to enable abbreviated dynamic protocols compatible with routine workflows [19,29,41,42,55,56,57,58,59]. In pediatric imaging, LAFOV PET/CT allows substantial dose reduction and shorter acquisitions, minimizing radiation burden and the need for sedation. In obese patients, the improved sensitivity and TOF performance enhance lesion detectability despite increased attenuation [13]. Similarly, oncology protocols increasingly employ shortened whole-body Patlak acquisitions to optimize the balance between quantitative precision and clinical practicality [44].

4. Therapy Monitoring and Response Assessment

Dynamic PET/CT is particularly valuable in monitoring therapy response, offering quantitative metrics that can detect changes earlier than traditional anatomical imaging and without being confounded, unlike static SUV scans, by the measurement time window relative to injection, inaccuracies in the amount of administered dose entering the arterial blood circulation and the body mass index of patients [60]. Parameters such as blood flow, glucose metabolism, and metabolic influx and efflux rate provide direct and quantitative measures of multiple aspects of cellular uptake activity, allowing clinicians to more reliably evaluate treatment efficacy at a molecular level [61].

This capability is crucial for assessing novel therapies, including immunotherapies and targeted treatments, where metabolic changes often precede anatomical responses and which can often be confounded by non-specific inflammatory reactions. For instance, a decrease in tumor glucose metabolic rate following treatment with a checkpoint inhibitor may indicate an effective immune response, even before tumor shrinkage is visible on CT or MRI [62] and which can be differentiated from normal tissue inflammation. This early feedback helps guide treatment decisions, optimizing outcomes for patients [47].

WBD-PET enables continuous, time-resolved quantification of radiotracer activity throughout the entire body, providing complete time–activity curves (TACs) for all organs at risk and target volumes [63]. This comprehensive temporal sampling reduces the uncertainty associated with extrapolation and interpolation that is inherent in sparse static sampling protocols, where activity concentrations are measured at only a few discrete time points. By capturing the true kinetics of tracer uptake and washout, WBD-PET allows for a more accurate numerical integration of TACs, resulting in more reliable and patient-specific residence time estimates. This, in turn, improves the accuracy of absorbed dose calculations and supports more individualized and optimized radionuclide therapy [42].

Dynamic imaging also supports adaptive therapy strategies, where treatments are tailored based on the patient’s ongoing response. By providing real-time insights into tumor biology, dynamic PET/CT facilitates a more personalized approach to cancer care [63].

5. Research Applications

The integration of whole-body dynamic PET/CT into research has opened new frontiers in understanding human physiology and disease. One of its primary applications is in the pharmacokinetic evaluation of new radiotracers, where dynamic imaging provides detailed data on tracer distribution, metabolism, and clearance. This information is invaluable for developing novel imaging agents and therapeutic compounds [64].

Beyond pharmacokinetics, dynamic PET/CT is instrumental in studying systemic pathophysiology. By capturing tracer kinetics across the entire body, researchers can investigate complex interactions between organs, such as the interplay between metabolic activity in the brain and heart during stress or disease states [65]. This holistic approach is also aiding the development of precision medicine, where therapies are tailored based on an individual’s unique physiological and molecular profile. Dynamic whole-body PET measurements are also crucial for accurate dosimetry of the targeted radionuclide agent throughout the body across both targeted and normal tissues to ensure both safe and efficacious personalized radionuclide treatment schemes [38].

PET imaging systems have been the cornerstone of molecular imaging for decades. However, they were traditionally limited to either static whole-body scans or dynamic limited-body section scans due to their limited sensitivity and axial FOV [28]. Nevertheless, modern PET/CT systems now enable, thanks to their TOF-enhanced sensitivity and/or longer AFOVs, both dynamic scans and whole-body coverage at the same time while supporting easy-to-use automated parametric imaging tools promising to transform clinical workflows and research potential [17].

Although high-TOF SAFOV PET systems can nowadays support dynamic multi-parametric whole-body imaging applications, it has been LAFOV scanners that have mostly driven this paradigm shift from static semi-quantitative to dynamic highly-quantitative multi-parametric PET imaging and the clinical adoption of this technology, thanks to their higher sensitivity, enhanced temporal resolution, population-based input function models to reduce scan times and their capability to complete dynamic whole-body scans at a single bed position for streamlined implementation of those scan protocols [66].

## 4. Challenges and Limitations

Dynamic PET/CT imaging offers profound insights into tracer kinetics and physiological processes but faces significant challenges in data acquisition and analysis. The technique involves continuous or segmented imaging over time, resulting in large datasets that require advanced computational tools for storage, processing, and interpretation [67]. The complexity of kinetic modeling further demands specialized expertise to extract quantitative parameters such as blood flow, metabolic rates, and receptor binding potentials. Variability in protocols and reconstruction techniques across different systems complicates standardization and reproducibility, limiting the integration of dynamic imaging into routine clinical workflows [68].

Another critical limitation is the prolonged scan time required for dynamic imaging, which can affect patient compliance and increase the risk of motion artifacts, such as respiratory or involuntary movement. These artifacts reduce image quality and complicate data analysis, particularly in whole-body dynamic PET/CT scans. The extended scan times also decrease patient throughput, posing logistical challenges in busy clinical settings [69]. Additionally, while high-sensitivity detectors in modern scanners mitigate some of these issues, dynamic imaging remains resource-intensive, requiring dedicated time and expertise [70]. The combined use of robust graphical analysis methods that employ linearized model equations, such as the Patlak method, represents a promising approach. When integrated with population-based input function models, it enables the generation of highly quantitative maps of macro-kinetic parameters. Importantly, this can be achieved using shorter scan times comparable to those used in conventional static PET imaging [19,42,54,55,56,57,58].

Although dynamic PET/CT offers significant quantitative advantages, its superiority over conventional static imaging is not universal across all clinical scenarios [47]. In inflammatory diseases, low-grade malignancies, or lesions with heterogeneous tracer kinetics, dynamic parameters may not consistently provide superior diagnostic performance compared to conventional SUV-based assessment [25]. In addition, parametric imaging may be affected by inaccuracies in image-derived or population-based input functions, patient motion, low count statistics, and mismatch between the selected kinetic model and the true biological tracer behavior, potentially resulting in unstable parameter estimates or misleading parametric maps [44].

Similarly, AI-assisted denoising and direct parametric reconstruction methods may introduce over-smoothing or artificial image features if not rigorously validated, raising concerns regarding clinical interpretation. These limitations emphasize that dynamic PET/CT should be applied selectively, where its additional quantitative value clearly outweighs the increased complexity, acquisition time, and computational demands [62].

Cost and accessibility further restrict the adoption of dynamic PET/CT. Long-axial-field-of-view (LAFOV) scanners, which are essential for efficient whole-body dynamic imaging, are expensive to acquire and maintain. This makes technology less accessible, especially in resource-limited settings [13]. Furthermore, the need for trained personnel to perform and interpret dynamic studies creates a barrier to widespread implementation. Despite these challenges, ongoing advancements in TOF detectors and cost-effective hardware configurations [17,71,72,73], direct 4D reconstruction methods and automated parametric imaging approaches, as described in previous studies by Karakatsanis et al. and Zhang et al. [15,60], and artificial intelligence tools [60,74] are addressing many of these limitations, paving the way for broader clinical adoption of dynamic PET/CT imaging [12].

An additional challenge is the environmental impact of producing radiotracers, which requires facilities with specialized infrastructure. Efforts to develop more efficient synthesis methods and sustainable practices are ongoing [11]. Variability in scanner performance between manufacturers also poses standardization challenges.

## 5. Future Directions

The future of whole-body dynamic PET/CT (WBD-PET/CT) is expected to be shaped less by the introduction of entirely new scanner concepts and more by the clinical translation, standardization, and validation of technologies that are already emerging in current practice [60]. Long-axial-field-of-view (LAFOV) PET/CT scanners, silicon photomultiplier-based detectors, time-of-flight reconstruction, and automated parametric imaging have already demonstrated the ability to support whole-body or near whole-body dynamic acquisitions with improved sensitivity, reduced scan duration, lower injected activity, and simultaneous kinetic assessment across multiple organs [72]. Therefore, the next major step will be the development of robust, reproducible, and indication-specific clinical workflows that can integrate WBD-PET/CT into routine imaging without substantially increasing patient burden, scanner occupancy, or interpretation complexity [71]. Shortened dynamic protocols based on population-based or image-derived input functions have already shown that clinically feasible 20–30 min dynamic FDG PET acquisitions can generate multiparametric images with acceptable quantitative performance, suggesting that future work should focus on multicenter validation, harmonization across vendors, and tracer-specific optimization rather than simply proving technical feasibility. Recent studies have shown that 20-min dynamic whole-body FDG PET using scaled population-based input functions can provide multiparametric imaging without compromising image quality or precision, while other LAFOV studies reported substantial scan-time reductions for Patlak Ki imaging with limited loss of quantitative accuracy [19,42,56].

A key future direction will be the definition of clinically actionable kinetic biomarkers. Although SUV-based PET/CT remains practical and widely established, WBD-PET/CT can provide complementary parameters such as net influx rate, distribution volume, perfusion-related indices, receptor-binding metrics, washout rates, and voxel-wise parametric maps [18]. These parameters may improve lesion characterization, therapy response assessment, systemic disease evaluation, and theranostic dosimetry, but their clinical value must be demonstrated against established endpoints, including diagnostic accuracy, prognostic stratification, treatment selection, patient outcome, and cost-effectiveness [38,43,75]. Dynamic and delayed total-body PET studies already suggest that parametric imaging can increase lesion conspicuity by separating biologically relevant tracer uptake components from nonspecific background signal; however, implementation remains limited by acquisition complexity, long protocols, and the need for standardized interpretation criteria. Future research should therefore prioritize disease-specific evidence, including oncology, neuroendocrine tumors, cardiovascular inflammation, infection/inflammation imaging, and radionuclide therapy planning, where kinetic information is most likely to alter clinical decision-making [66,76].

Artificial intelligence will also have an important but more targeted role in the future of WBD-PET/CT. Rather than being presented as a stand-alone solution, AI should be considered an enabling tool for motion correction, image reconstruction, denoising, segmentation, kinetic modeling, quality control, and decision support [77]. AI-based denoising and reconstruction methods are already being evaluated for reducing scan duration or injected dose, and recent work has shown measurable improvements in image quality metrics for short-acquisition PET data [68]. Nevertheless, their future clinical use will depend on transparent training datasets, external validation, uncertainty estimation, preservation of quantitative accuracy, and safeguards against over-smoothing or generating features not supported by the measured PET data. This is particularly important in parametric WBD-PET/CT, where small changes in time-activity curves may influence kinetic biomarkers and subsequent clinical interpretation [18,54,60].

Finally, WBD-PET/CT is likely to become increasingly important in theranostics and personalized medicine. Its ability to measure tracer kinetics across tumors and normal organs may support more accurate patient-specific dosimetry, individualized treatment planning, early therapy response assessment, and adaptive radionuclide treatment strategies [54,56]. Novel radiotracers targeting specific biological pathways, together with simplified dynamic acquisition schemes and automated multiparametric reconstruction, may broaden the clinical role of WBD-PET/CT beyond FDG oncology toward receptor imaging, immune-oncology, inflammation, neurology, cardiology, and systemic multi-organ disease [41,58]. Digital-twin approaches and predictive modeling remain promising but should currently be framed as exploratory and translational rather than established clinical tools. Overall, the future of WBD-PET/CT will depend on transforming its quantitative potential into standardized, interpretable, and clinically actionable information that improves patient management while remaining practical for routine nuclear medicine workflows [2,24,78].

## 6. Conclusions

Whole-body dynamic PET/CT represents a transformative advancement in molecular imaging, bridging the gap between static semi-quantitative imaging and fully quantitative, multi-parametric assessment of physiological processes. Enabled by innovations in detector technology, long axial field-of-view systems, and advanced reconstruction and kinetic modeling techniques, WBD-PET/CT provides enhanced diagnostic accuracy, improved lesion characterization, and more reliable treatment monitoring.

Despite these advantages, several challenges remain, including increased data complexity, the need for standardized acquisition and analysis protocols, higher costs, and the requirement for specialized expertise. Addressing these limitations will be essential for the broader clinical adoption of this technology.

Looking forward, ongoing developments in artificial intelligence, direct parametric imaging, digital twin modeling, and novel radiotracers are expected to further expand the capabilities of WBD-PET/CT. These advancements will support more personalized, data-driven diagnostic and theranostic approaches, ultimately improving patient outcomes and advancing precision medicine.

## Data Availability

No new data were created or analyzed in this study.
